# The role of necroptosis in common respiratory diseases in children

**DOI:** 10.3389/fped.2022.945175

**Published:** 2022-07-25

**Authors:** Junjie Ning, Lina Qiao

**Affiliations:** ^1^Pediatric Intensive Care Unit, West China Second University Hospital, Sichuan University, Chengdu, China; ^2^Key Laboratory of Birth Defects and Related Diseases of Women and Children, Ministry of Education, Sichuan University, Chengdu, China; ^3^NHC Key Laboratory of Chronobiology, Sichuan University, Chengdu, China

**Keywords:** necroptosis, RIPK1, RIPK3, MLKL, Necrostatin-1

## Abstract

Studies have shown that necroptosis (NEC) relies on a unique gene-regulated molecular pathway to cause cell death. With the development of knockout mouse models and specific molecular inhibitors of necrotic proteins, this cell death pathway has been considered one of the important causes of the pathogenesis of human diseases. In this review, we explored the possible roles and mechanisms of NEC in common respiratory diseases in children, such as acute lung injury, acute respiratory distress syndrome, pulmonary infection, childhood asthma, pulmonary hypertension, etc., in order to provide new ideas for the prevention and treatment of such diseases.

## Introduction

Each type of cell death can exhibit unique morphological characteristics. Using these features, cell death can be divided into three categories, i.e., necrosis, apoptosis, and autophagy, which is also referred to as the regulatory cell death (RCD) pathway. The traditional concept of necrosis is passive cell death resulting from the accidental death of cells caused by damage in an extreme microenvironment. Apoptosis is highly regulated programmed cell death; it is a non-lytic and usually non-immunogenic form of cell death. Autophagy is a self-protective regulatory means of decomposing and eliminating unnecessary or dysfunctional components in cells. In recent years, scholars have proposed a new type of cell death: necroptosis (NEC). The morphology of necroptotic cells is similar to that of necrotic cells; however, unlike necrosis, NEC is regulated by internal mechanisms. NEC is actively regulated and controlled by the intracellular receptor-interacting serine/threonine-protein kinase 1 (RIPK1)/RIPK3/mixed-lineage kinase domain-like protein (MLKL) signaling pathway (1), which induces orderly and regular cell death; this process is lytic and highly inflammatory. With the development of knockout mouse models and specific molecular inhibitors of necrotic proteins, researchers have determined that NEC is present in almost all tissues and diseases. Currently, NEC is considered to play a key role in the regulation of various physiological processes, including acute and chronic neurodegenerative diseases such as Parkinson’s disease and Alzheimer’s disease (2), tumor immunity and metastasis (3), and infectious diseases such as influenza (4). However, only recently has the relationship between NEC and lung diseases such as chronic obstructive pulmonary disease, asthma, idiopathic pulmonary fibrosis, and acute respiratory distress syndrome (ARDS) in adults been investigated (5). In this manuscript, we review abnormal RIP1/RIP3/MLKL pathway activation and the mechanisms of action during the pathogenesis of lung diseases in children and provide new ideas for the prevention and treatment of NEC in children with lung diseases.

## Mechanisms of necroptosis and lung diseases

**FIGURE 1:**
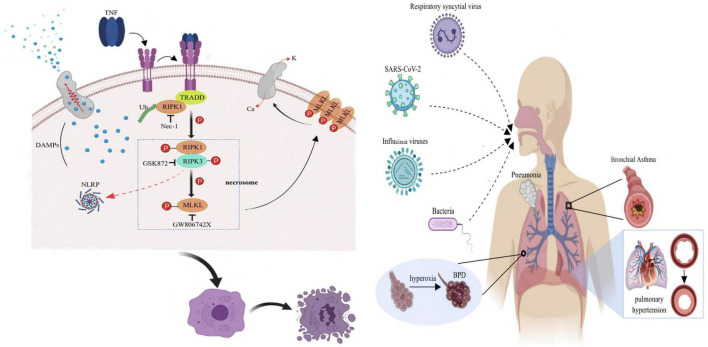
Schematic diagram of the molecular mechanism of TNF-α-mediated NEC.

Necroptosis is a cellular response to environmental stress. These environmental stresses can be caused by chemical or mechanical damage, inflammation or infection (6), but the specific mechanisms have not been elucidated. One current proposal is that NEC is mainly triggered by tumor necrosis factor receptor 1 (TNFR1), Toll-like receptor 4 (TLR4), and Z-DNA binding protein 1 (ZBP1). The mechanism of action of each is similar. In this manuscript, TNFR1 is used as an example to introduce the signal transduction process (Figure 1). After TNFR1 binds to its ligand, i.e., tumor necrosis factor-α (TNF-α), its intracellular death-fold motif is exposed, and downstream molecules such as RIPK1 are recruited, forming a complex that consists of TNFR1-associated death domain protein (TRADD), RIPK1, and linear ubiquitin chain assembly complex (LUBAC) and further forming a necrotic body that is composed of RIPK1, RIPK3, and MLKL. RIPK3 is activated by RIPK1, and the 2 catalyze a complex series of phosphorylation reactions through RIP isoform motif interactions, among which RIPK3 catalyzes MLKL phosphorylation to form MLKL multimers (trimers or tetramers). MLKL executes NEC, and its multimers promote extracellular Ca^2+^ inward flow and intracellular K^+^ outward flow and increase the permeability of the cell membranes to water by binding to phosphatidylinositol phosphate esters, ultimately causing cell swelling, disintegration and death (7). Second, the formation of pores can also lead to the release of intracellular damage-associated molecular pattern (DAMP) contents (such as HMGB1, mitochondrial DNA, uric acid, histones, and ATP) outside the cells, further aggravating the inflammatory response (8). In summary, RIPK1/RIPK3/MLKL is the classical NEC signal transduction pathway, of which RIPK3 and MLKL are the key effectors of injury propagation (9). In addition, RIPK3 regulates the transcription of proinflammatory cytokines and activate inflammasomes such as NLRP to aggravate tissue damage without the involvement of MLKL (10). With a deepening understanding of the mechanism of NEC, increasing evidence indicates that NEC is one of the driving factors in the development of lung injury and is involved in the pathogenesis of a variety of lung diseases such as chronic obstructive pulmonary disease, asthma, idiopathic pulmonary fibrosis, and ARDS (5, 11).

### Necroptosis and acute lung injury

Acute lung injury is a clinical disease with high morbidity and high mortality induced by a variety of factors. In addition to direct damage to alveolar structures caused by physical damage induced by infection, hyperoxia, and mechanical ventilation (MV), the dysregulation of the inflammatory response is also an important cause of ALI, and an excessive inflammatory response eventually leads to diffuse damage to the alveolar capillary membrane and hyperpermeable pulmonary edema. Currently, it is believed that ALI is caused by an inflammatory cascade, oxidative stress responses, or cell death. However, its pathogenesis is still not completely clear. Recent studies have shown that NEC plays an important role in this process. Chen et al. (10) observed a significant upregulation of necrosis and the NLRP3 inflammasome pathway in the lungs of lipopolysaccharide (LPS)-induced ALI mouse. The selective RIP3 inhibitor GSK872 significantly inhibited necrosis and NLRP3 activation and reduced the production of IL-1β and IL-18 and the infiltration of inflammatory cells, significantly improving lung injury. Li et al. (12) found that the necrosis-related markers RIP1, RIP3, and MLKL were significantly increased in a neonatal mouse model of ALI induced by LPS exposure. A lack of RIPK3 can reduce the expression of cytokines and alleviate the ALI caused by the H7N9 influenza virus in mice (13). The above animal experiments all revealed that NEC plays an important role in inflammation/sepsis-mediated ALI.

Long-term exposure to hyperoxia causes lung tissue damage, an effect that is generally considered to be related to oxidative stress caused by intracellular reactive oxygen species (ROS), and a secondary inflammatory response of the host exacerbates lung injury during the process. Preterm infants are less resistant to oxidative stress and are more susceptible to hyperoxia acute lung injury (HALI). Oxidative stress disrupts lung development mainly through mechanisms that affect growth factor signal transduction, extracellular matrix assembly, cell proliferation, apoptosis, and angiogenesis. However, all these pathological changes are mainly concentrated in one central event: alveolar cell death (14). This cell death is primarily NEC (15). The results of an animal experiment showed that the lung pathology of hyperoxic rats after pretreatment with necrostatin-1 (Nec-1) significantly improved, suggesting that necrosis is related to lung injury caused by hyperoxia exposure. Western blot and RT–PCR were used to further detect the expression of key proteins and mRNAs, respectively, in the NEC pathway, and the interaction between RIP1 and RIP3 was evaluated by immunoprecipitation. It was found that hyperoxia exposure significantly increased the levels of protein and mRNA expression of RIP1, RIP3, and MLKL and enhanced the interaction between RIP1 and RIP3 in the lung (16), further indicating that hyperoxia can activate NEC.

Mechanical ventilation is an important means of life support for critically ill patients. It is often used to improve hypoxemia in ALI patients. However, improper MV use can also cause the excessive expansion of lung tissues and local atelectasis, inducing and aggravating inflammatory responses. Pressure-induced mechanical injury caused by MV and a hyperoxic environment can both cause airway and alveolar epithelial cell NEC and induce ventilator-induced lung injury (VILI). Through a prospective cohort study, Siempos et al. (17) compared the plasma levels of RIPK3 in MV patients and non-MV patients and found that there were significant differences between the two groups; however, there were no significant differences in plasma RIPK1 and MLKL concentrations between the two groups. In a study that used a mouse model of injury-induced MV with a high tidal volume of 25 mL/kg, Ripk3−/− mice were protected from lung inflammation and injury; similar results were not observed in RIPK1± and MLKL−/− mice after Nec-1 intervention. Therefore, the classical NEC signaling pathway, i.e., RIPK1/RIPK3/MLKL, may not be involved in the pathological process of VILI, but RIPK3 still plays an important role. This suggests that RIPK3 may mediate some inflammatory signaling pathways without the involvement of RIPK1 or MLKL.

Ischemic/reperfusion (I/R) injury refers to tissue damage caused by blood reperfusion after a period of hypoxia or ischemia (18) resulting from a variety of clinical diseases, including myocardial infarction, cerebral infarction, and gastrointestinal tract dysfunction. The pathophysiology of I/R injury is divided into two stages. First, the ischemic process leads to cell damage and hypoxia, which induce oxidative stress and subsequent inflammatory responses. Then, when the reperfusion phase begins, the activated endothelial cells produce excessive amounts of ROS, which further cause oxidative stress and inflammatory damage, eventually leading to apoptosis, autophagy, and necrosis (19). It is currently believed that the mechanism of pulmonary parenchymal I/R injury is similar to that of reperfusion injury in other organs, a process that includes a significant increase in ROS, intracellular calcium influx, endothelial cell injury, complement system activation, and the release of inflammatory mediators such as tumor necrosis factor receptor-related factors (20, 21). It has been shown that Nec-1 treatment significantly inhibits IR-induced necrotic cell death and the production of mitochondrial ROS (22). Thus, it is possible that NEC is associated with lung I/R injury. However, further studies are needed to clarify this hypothesis.

### Necroptosis and acute respiratory distress syndrome

Acute respiratory distress syndrome is an inflammatory pulmonary disease with a high mortality rate. It causes tissue hypoxia by interfering with the passage of oxygen from the alveoli to the blood. Previous studies have suggested that apoptosis is the only form of cell death that can be regulated. Therefore, apoptosis has been extensively studied in the occurrence and development of ARDS. A large number of reports have also shown that apoptosis is involved in the cell death pathway of ARDS (23). However, unlike apoptosis and autophagy, NEC is characterized by the release of DAMPs to the extracellular environment through cell lysis, thereby triggering inflammation. Because inflammatory responses play an important role in ARDS, NEC may accelerate tissue damage through proinflammatory responses. Pan et al. (24) found that RIPK1, RIPK3, and MLKL were expressed in large amounts in the lung tissue of oleic acid-induced ARDS rats and that Nec-1 pretreatment reduced the RIPK1-RIPK3 interaction, downregulated the RIPK1-RIPK3-MLKL signaling pathway, and inhibited inflammatory responses by reducing neutrophil infiltration and protein leakage into lung tissues, significantly improving lung function and reducing pulmonary edema in oleic acid-induced ARDS rats. Yu et al. (25) found that RIPK3 protein levels were significantly increased in the plasma and bronchoalveolar lavage fluid of patients with ARDS. Furthermore, in a high-dose LPS-induced severe ARDS mouse model, lung injury was mainly caused by rip3-mlkl-mediated NEC and endothelial dysfunction. The results reported by Wang et al. (26) also indicated that rip3-mediated NEC was the main mechanism of lung injury in high-dose LPS-induced ARDS mice. A cohort study showed that the amount of change in plasma RIPK3 from presentation to 48 h was independently associated with the occurrence of ARDS. The increase in plasma RIPK3 concentration in patients with ARDS 6 days before onset was significantly greater than that in patients without ARDS; the amount of change in plasma RIPK3 was also positively correlated with the 30-day mortality rate (27). Notably, the concentration of RIPK3 was also increased in exosomes in the alveolar lavage fluid of ARDS patients (28).

Massive blood transfusion is one of the most common risk factors for ARDS, but the mechanism of blood transfusion-related ARDS is still unclear. Through *in vitro* experiments, researchers found that human pulmonary microvascular endothelial cells (ECs) released RIPK3 and HMGB1 after incubation with erythrocytes and that Nec-1 treatment reduced the death of ECs and reduced the release of RIPK3 and HMGB1. In a mouse blood transfusion model, blood transfusion increased the formation of pulmonary necrosomes and the release of HMGB1 and simultaneously increased the sensitivity of mice to LPS-induced pulmonary inflammation. The above results suggest that NEC may be the mechanism by which ARDS develops after a blood transfusion (29).

### Necroptosis and pulmonary infection

Studies have found that NEC participates not only in some necrotizing diseases, especially in the physiological regulation of infectious diseases, but also in their occurrence, development, and prognosis. For example, during a viral infection, the virus releases nucleic acids into cells and activates the endogenous NEC pathway, which effectively inhibits the transmission of intracellular pathogens, thereby preventing viral replication. The process may be beneficial to the host/organism. However, it is characterized by cell rupture and the release of intracellular immunogenic contents. These contents eventually trigger the body’s inflammatory response. The lung is one of the most important target organs for the secretion and release of proinflammatory mediators during sepsis and trauma. Once the cytokine immune balance is disrupted, the uncontrolled release of inflammatory mediators occurs, resulting in lung tissue damage (30).

Pneumonia pathogens, such as influenza A virus (31), Streptococcus pneumoniae, and Staphylococcus aureus, as well as pathogens that cause health care-associated pneumonia, such as carbapenem-resistant Klebsiella pneumoniae and Serratia marcescens, have been shown to activate cell death through the RIPK1/RIPK3/MLKL cascade, leading to lung parenchymal necrosis. In addition, the activation of this pathway leads to the development of NEC in specific types of immune cells, especially macrophages, contributing to pathogenesis by depriving the host of critical immunomodulatory functions (32). Severe acute respiratory syndrome coronavirus 2 (SARS-CoV-2) is the main pathogen of coronavirus disease 2019 (COVID-19) and can cause respiratory diseases and multiple organ failure in severely ill patients. Although virus-induced lung injury and inflammatory cytokine storms are considered to be directly related to the clinical manifestations of COVID-19, the underlying mechanism that triggers the inflammatory response remains unclear. Karki et al. (33) found that TNF-α and IFN-γ cotreatment activated the JAK/STAT1/IRF1 axis to induce nitric oxide production and drive caspase-8/FADD-mediated PANoptosis, which is characterized by gasdermin-mediated pyroptosis, caspase-8-mediated apoptosis, and MLKL-mediated NEC. The single loss of pyroptosis, apoptosis, or necrosis mediators is not sufficient to protect cells from death; in contrast, cells with the simultaneous loss of RIPK3 and caspase-8 or RIPK3 and FADD are resistant to this type of cell death.

Respiratory syncytial virus (RSV) is the main cause of acute bronchiolitis in children under 2 years of age. It has a high prevalence and is one of the main causes of mortality in infants. Its pathological feature is the death of airway epithelial cells (AECs). However, the manner of AEC death is still unknown. Through experiments with RSV-infected mice, Santos et al. (34) found that tumor necrosis factor-mediated RSV triggers NEC in macrophages. The NEC pathway is also involved in TNF secretion, which further aggravates lung injury during RSV infection. The expression of the Ripk3 and Mlkl genes in Tnfr1−/− mice was significantly reduced, and the number of alveolar macrophages in the lungs was also drastically reduced. In summary, RSV infection triggers macrophage NEC in mice in a RIPK1-, RIPK3-, and MLKL-dependent manner, and this type of NEC is not conducive to virus clearance. In addition, Simpson et al. (35) used RSV to inoculate primary human AECs and found that HMGB1 levels were increased in nasopharyngeal specimens from children with RVS infection and that RSV induced epithelial cell death and increased phosphorylated RIPK1 and MLKL levels, whereas RIPK1 or MLKL inhibition attenuated the RSV-induced translocation and release of HMGB1 and reduced the viral load, further indicating that RSV is involved in the pathogenesis of bronchiolitis through NEC.

Tuberculosis is still an important infectious disease that endangers human health. According to the latest statistics of the World Health Organization (WHO), approximately 9.9 million people worldwide had tuberculosis in 2020, of whom 11% were children (36). Macrophages are the first line of defense encountered by Mycobacterium tuberculosis (Mtb) after entering the lungs. After bacteria invade the body, most will be engulfed by macrophages. However, recent studies have found that Mtb can escape from phagosomes and induce the leakage of bactericidal substances, such as ROS and reactive nitrogen species (RNS), and lysosomal membrane permeabilization (LMP), resulting in cell injury and potentially leading to cell death (37–40). The results from recent studies suggest that this cell death mechanism is mainly due to the excessive production of TNF-α in Mtb-infected macrophages, thus activating the RIPK1-MLKL pathway and leading to the rapid NEC of macrophages. In addition, in Mtb-infected fibroblasts, excessive TNF-α will induce the continuous production of a large amount of ROS in mitochondria, quickly activating the NEC pathway, thus leading to cell lysis and the induction of immune pathological damage and thereby promoting the proliferation and spread of bacteria in the body, which is ultimately not conducive to infection control (41).

### Necroptosis and childhood asthma

Asthma is a chronic airway inflammatory disease characterized by increased eosinophils and lymphocytes, goblet cell metaplasia, smooth muscle activation, and airway hyperresponsiveness. Type II cytokines (e.g., IL-4, IL-5, and IL-13) secreted by activated Th2 cells play a role in the chronic airway inflammatory response in most asthma cases. The regulation of epithelial cell death has become a key mechanism for the control of barrier immune homeostasis. However, the role of RCD, especially NEC, in lung homeostasis and related pulmonary immune diseases is still poorly understood. Using house dust mite (HDM) extract allergens and a stimulation-induced asthma mouse model, the development of asthma pathology was prevented in mice by the inactivation of RIPK1 kinase expression and RIPK3 or MLKL deficiency (42). IL-33 is a proinflammatory cytokine that plays an important role in inflammatory diseases. After its release, IL-33 can activate immune cells to induce type 2 immune responses, which are the initial link of the immune response in the development and progression of bronchial asthma. Although it is known that IL-33 is released during tissue injury, the exact release mechanism is still not fully understood. Shlomovitz et al. (43) found that, under physiological conditions, IL-33 is stored in the nucleus and that the release of IL-33 can be controlled through the RIPK3/MLKL and caspase-independent cell death pathways. Once IL-33 is passively released extracellularly, it acts as an alarmin, activating basophils and eosinophils to induce airway inflammation. RIPK3-MLKL-dependent NEC is believed to also cause eosinophil lysis and release eosinophil granules to increase airway reactivity (44). Simpson et al. (35) infected IRF7−/− mice with pneumonia virus of mice (PVM) in early life and treated them with Nec-1 or GW806742X (MLKL inhibitor) in infancy for 6 weeks. Subsequently, asthma was induced by PVM or exposure to cockroach extracts (once per week for 4 weeks). The results showed that asthma sensitivity was reduced in IRF7−/− mice by the drug inhibition of RIPK1- or MLKL-induced tracheal smooth muscle remodeling, mucus hypersecretion, and reduced eosinophilic inflammation.

### Necroptosis and pulmonary arterial hypertension

Pulmonary arterial hypertension is a serious disease characterized by perivascular inflammatory cell infiltration and pulmonary vascular remodeling, which eventually leads to right heart failure, in which inflammation and immunity play important roles in the pulmonary vascular remodeling and pathogenesis of PAH. However, how they promote pulmonary vascular remodeling and progression is not fully understood (45). Through animal experiments, Xiao et al. (46) found that the Toll-like receptor (TLR) and Nod-like receptor (NLR) pathways in PAH rats were activated and DAMPs were upregulated and that RIPK3-mediated NEC is involved in the production of DAMPs, ultimately leading to PAH. In addition, HMGB1, a DAMP, is a ubiquitous DNA-binding protein with extracellular proinflammatory activity. Once released from damaged cells, HMGB1 promotes inflammatory responses and tissue repair and promotes the proliferation of pulmonary artery smooth muscle cells and pulmonary ECs, triggering pulmonary vascular remodeling. HMGB1 has been considered a key factor in the development of PAH (47). Many studies have shown that HMGB1 can be released by human pulmonary artery ECs and human pulmonary artery smooth muscle cells through necrosis or NEC, which triggers a cascade of inflammatory responses (48, 49).

### Necroptosis and bronchopulmonary dysplasia

Different from ALI, BPD has a chronic course. Although the pathogenesis of the two diseases is not yet fully understood, a large number of studies have shown that there is a certain overlapping genetic background between the two diseases (50, 51), and both can develop and progress in response to adverse factors such as infection and inflammatory reactions. ALI and BPD may be the manifestation of the same disease at different stages. Studies based on bioinformatics analysis have found that the pathogenesis of BPD is complex and that many signal transduction pathways and genes are jointly involved in it. The pulmonary T cell receptor (52), nuclear factor-erythroid 2-related factor 2 (Nrf2) (53), mitogen-activated protein kinase (MAPK) (54), Keap-1/Nrf2 (55), and TGF β1/Smads (56) pathways may be involved in the development of the disease. However, the role of the RIP1/RIP3/MLKL pathway, the classical NEC signaling pathway, in BPD has not been described. Studies have found that TREM-1 can reduce lung injury in neonatal mice under hyperoxia exposure through the downregulation of RIPK3-mediated necrosis and ultimately reduce lung inflammation and improve alveolarization; furthermore, phosphorylated RIPK3 and MLKL protein expression was increased in the alveolar lavage fluid in infants with BPD (57). Therefore, we speculate that NEC mediated by the RIP1/RIP3/MLKL pathway may be involved in the occurrence and development of BPD, a hypothesis worthy of further study.

## Necroptosis inhibitors

Specific inhibitors for different targets, necroptosis inhibitors can be divided into RIPK1 inhibitors, RIPK3 inhibitors and MLKL inhibitors. Among the inhibitors of necroptosis, the Necrostatins family has the most relevant studies, among which Necrostatin-1 (Nec-1) is the most typical. The main structure of Nec-1 is a linker of indole and hydantoin, which belongs to alkaloids, and its molecular weight is 259.3D. As an effective specific inhibitor of necroptosis, this substance has the following characteristics: (1) Nec-1 can specifically inhibit the necroptosis pathway, but does not affect apoptosis and autophagy; (2) Nec-1 does not affect the normal physiological functions of normal cells, including cell proliferation and cycle distribution, cell membrane integrity, ATP level mitochondrial membrane potential, mRNA expression, etc.; (3) Allosteric effects of Nec-1 on the kinase activity of RIP1 Inhibition is through the combination of the activation loop (activation loop, also called T loop) between its N-terminus and C-terminus, which blocks the position of the reaction, so that the activity of the enzyme is lost, rather than affecting the role of other functional domains (58). A large number of research results have shown that Nec-1 has a significant protective effect on lung diseases such as acute lung disease and acute respiratory distress syndrome (24, 59, 60).

## Conclusion

The NEC cell death pathway has basically been established, with RIPK1, RIPK3, a mixed series of protein kinase-like domains and other related proteins playing important roles. Under normal conditions, NEC is beneficial for resisting the invasion of pathogenic microorganisms and maintaining a stable environment in the body. Studies have confirmed that NEC also plays a substantial role in the pathogenesis of lung diseases in children. Therefore, in-depth investigations of the regulatory mechanisms of NEC and the elucidation of the relationship between NEC and other types of cell death will help establish the specific role of NEC in lung diseases. NEC is not only an important supplement to the cell death system but also provides a new direction for the treatment of lung diseases. There are still many issues that need to be explored, for example, RIPK1 and RIPK3 genetic polymorphisms, the development of specific sensitive markers of NEC, and the use of NEC to effectively and feasibly treat the disease.

## Author contributions

JN: data collection, sorting and analysis, and manuscript writing. LQ: coaching. Both authors contributed to the article and approved the submitted version.
